# Interface Engineered V-Zn Hybrids: Electrocatalytic and Photocatalytic CO_2_ Reductions

**DOI:** 10.3390/nano12162758

**Published:** 2022-08-11

**Authors:** Seon Young Hwang, Hye Ji Jang, Young Jun Kim, Ju Young Maeng, Go Eun Park, Seo Young Yang, Choong Kyun Rhee, Youngku Sohn

**Affiliations:** 1 Department of Chemistry, Chungnam National University, Daejeon 34134, Korea; 2 Department of Chemical Engineering and Applied Chemistry, Chungnam National University, Daejeon 34134, Korea

**Keywords:** V-Zn hybrids, electrocatalytic CO_2_ reduction, photocatalytic CO_2_ reduction, formate, syngas, Fisher-Tropsch synthesis

## Abstract

V-Zn hybrids have widely been used as catalyst materials in the environment and as energy. Herein, V-Zn hybrid electrodes were prepared by the hydrothermal and sputter-deposition methods using a Zn foil support. Their electrocatalytic CO_2_ reduction (EC CO_2_ RR) performances were tested under various applied potentials, different electrolytes, and concentrations before and after thermal treatment of the demonstrated electrode. Gas and liquid products were confirmed by gas chromatography and nuclear magnetic resonance spectroscopy, respectively. For V-Zn electrode by hydrothermal method produced mainly syngas (CO and H_2_) with tunable ratio by varying applied potential. Minor products include CH_4_, C_2_H_4_, and C_2_H_6_. A liquid product of formate showed a Faradaic efficiency (FE) of 2%. EC CO_2_ RR efficiency for CO, CH_4_, and formate was best in 0.2 M KHCO_3_ electrolyte condition. CO and formate were further increased by photoirradiation and Nafion-treated electrode. Formate and CH_4_ productions were significantly increased by thermal treatment of the V-Zn electrode. CO production was diminished for the V-Zn electrode by sputter deposition but was recovered by thermal treatment. Photocatalytic CO_2_ RR was tested to find that RR products include CH_3_OH, CO, CH_4_, C_2_H_4_, and C_2_H_6_. Interestingly long-chain hydrocarbons (C_n_H_2n_ and C_n_H_2n+2_, where n = 3–6) were first observed under mild conditions. The long-chain formation was understood by Fisher-Tropsch (F-T) synthesis. Alkenes were observed to be more produced than alkanes unlike in the conventional F-T synthesis. The present new findings provide useful clues for the development of hybrid electro-and photo-catalysts tested under various experimental conditions in energy and environment.

## 1. Introduction

Hybrids of different metal elements and their oxides have extensively been employed in catalyst application areas for energy and environments [1,2,3,4,5]. Among them, V-Zn hybrids with diverse compositions and morphologies have been developed and applied to photocatalysts, electrocatalysts, and energy storage materials [6,7,8,9,10,11,12,13,14,15,16,17,18]. In photocatalytic catalyst application of a V-Zn hybrid, it was theoretically found that photogenerated carriers are efficiently separated at the interface of V_2_O_5_/ZnV_2_O_6_, where the O 2p state is highly contributed near the Fermi level in the valence band structure [13]. Li et al. synthesized V_2_O_5_/ZnV_2_O_6_ nanosheets by the solvothermal method and the post thermal treatment (300–500 °C) [14]; they tested photocatalytic CO_2_ reduction activities for V_2_O_5_/ZnV_2_O_6_ nanosheets, V_2_O_5_, and ZnV_2_O_6_, and found that the V_2_O_5_/ZnV_2_O_6_ showed the highest CO production activity; 2.2× and 1.9× higher than those observed in V_2_O_5_ and ZnV_2_O_6_, respectively. The enhanced activity was attributed to charge transfer from ZnV_2_O_6_ to V_2_O_5_ at the interface and enhanced adsorption of CO_2_ on the surface. Similarly, in the photocatalytic CO_2_ RR over ZnV_2_O_6_/g-C_3_N_4_, CH_3_OH, CO, and CH_4_ were reported to be more produced than in ZnV_2_O_6_ and g-C_3_N_4_, attributed to the synergistic interfacial hybrid effect [15]. In the case of photocatalytic CO_2_ RR over ZnO, V_2_O_5_, ZnO/V_2_O_5_ composite, and ZnV_2_O_6_ nanosheets [16], Bafaqueer et al. reported that ZnV_2_O_6_ nanosheets showed the highest performance on CH_3_OH production by CO_2_ RR. The activity of ZnV_2_O_6_ was 3.4× higher than that of the ZnO/V_2_O_5_ composite. In this experiment, formic acid and acetic acid were newly reported; they also found that the CO_2_ RR activity was poorly degraded upon calcination at 550 °C; this indicates that many factors are involved in the mechanism, including surface and interface engineering. Tahir also reported a similar observation that hierarchical 3D ZnV_2_O_4_ microspheres showed higher CO_2_ RR performance than ZnO/V_2_O_5_ composite [17]. In this experiment, CO_2_ RR products include CO, CH_3_OH, CH_4_, and C_2_H_6_. As discussed above, the interface plays a significant role in CO_2_ RR efficiency and it needs to be further elucidated in other developed catalyst systems.

Although there are several works of literature on the photocatalytic CO_2_ RR [11,12,13,14,15,16,17] no studies have been reported on EC CO_2_ RR over V-Zn hybrids although it shows electrochemical activity on water oxidation and hydrogen production [18,19]. From a practical point of view, EC CO_2_ RR is more feasible in the production of value-added products from CO_2_ than photocatalytic CO_2_ RR [20]. In EC CO_2_ RR over V-Zn hybrids and their oxides, CO and H_2_ (called syngas) are expected to be produced because Zn is known to produce CO by EC CO_2_ RR [3,4,21,22,23] and the Zn-V hybrid has shown EC activity on hydrogen production [19]. Guzman et al. prepared CuZnAl-oxide nanomaterials and demonstrated that H_2_/CO ratio could be tuned by applying potential [24]. Therefore, the syngas production performance needs to be tested for V-Zn hybrids. CH_4_, formate/formic acid, alcohols, and multi-carbon (C_n≥2_) products are also included in CO_2_ reduction products [3,4,25,26,27,28,29]. Therefore, EC CO_2_ RR products and efficiencies are needed to be newly examined by modifying the V-Zn interface.

Motivated by this and the potential application of V-Zn hybrids to EC CO_2_ RR, we have directly prepared diverse interface-engineered V/Zn electrodes using Zn foil support. The hydrothermal method and sputter-deposition method were both employed, and the prepared electrodes were tested before and after thermal annealing. Zn sputter deposition was also performed on V support to examine the difference between V/Zn and Zn/V before and after thermal treatment. On the basis of CO_2_ RR products and Faradaic efficiencies tested over the systematically prepared electrodes, the roles of the V/Zn interface were further deeply discussed. The oxidation states of the diverse V-Zn hybrid electrodes were examined by X-ray photoelectron spectroscopy (XPS) before and after EC CO_2_ RR. The stability of the electrodes was found to be dependent on the preparation methods. Thus, the present unique results provide clues on the interface engineering of V-Zn hybrids for improving CO_2_ RR performance and stability, and strategy for the development of hybrid catalysts for energy and environment.

## 2. Materials and Methods

For the preparation of V-Zn hybrids by the hydrothermal method, a Zn foil (30 mm × 5 mm, 2 mm thick) was polished and cleaned by sonication in deionized water. A solution of 1 mM vanadium (V) was prepared by dissolving Na_3_VO_4_ (99.9%, Thermo Scientific, Waltham, MA, USA) in deionized water. 60 mL of 1 mM V solution was taken into a 100 mL-size Teflon-lined stainless autoclave and a pre-cleaned Zn foil was dipped in the solution. After that, the autoclave was tightly capped and placed in an oven set at 180 °C for 24 h. After completion of the reaction, and the autoclave was naturally cooled to laboratory temperature the Zn foil was removed, cleaned with deionized water, and dried under an infrared lamp. The V-Zn hybrids prepared by the hydrothermal method were abbreviated as V-Zn(H). The V-Zn(H) sample was thermally treated at 400 °C for 1 h, and abbreviated as V-Zn(H-h). Vanadium (V) was sputter-deposited on a Zn foil support using an SPT-20 ion sputter coater (COXEM Co., Daejeon, Korea) at an ionization current of 5 mA for 600 s and 2400 s, and abbreviated as V(600 s)/Zn(S) and V(2400 s)/Zn(S), respectively. Their thermal treated (at 400 °C for 1 h) samples were abbreviated as V(600 s)/Zn(S-h) and V(2400 s)/Zn(S-h), respectively. Zn was also sputter-deposited on a V foil support at 5 mA for 600 s and 2400 s, and abbreviated as Zn(600 s)/V(S) and Zn(2400 s)/V(S), respectively. Their thermal treated (at 400 °C for 1 h) samples were abbreviated as Zn(600 s)/V(S-h) and Zn(2400 s)/V(S-h), respectively.

The crystal phases of the V-Zn(H) sample were examined using a X-ray (Cu K_α_ radiation) diffractometer (MiniFlex II, Rigaku Corp., Tokyo, Japan) in CNU Chemistry Core Facility. The surface morphologies of the demonstrated samples were examined before and after EC CO_2_ RR using a scanning electron microscope (SEM, model S-4800, Hitachi Ltd., Tokyo, Japan) setting at 10.0 keV. For Raman spectra of V-Zn(H) sample before and after EC CO_2_ RR, a LabRAM HR-800 UV-Visible-NIR Raman spectrometer (Horiba Jobin Yvon, Kyoto, Japan) was used with experimental conditions of 514 nm laser light, a 100× objective, and the monochromator 1800 grating. For UV–visible absorption spectrum for V-Zn(H), a NeoSys-2000 double beam UV–visible spectrophotometer (SCINCO Co., Ltd., Seoul, Korea) was used with a diffuse reflectance mode. The oxidation states and the surface compositions of the demonstrated electrodes before and after EC CO_2_ RR were examined by taking X-ray photoelectron spectra (XPS) using a K-Alpha^+^ XPS spectrometer (Thermo-VG Scientific, Waltham, MA, USA) equipped with a hemispherical energy analyzer and a monochromated Al Kα X-ray (1486.6 eV) light.

Electrocatalytic CO_2_ reduction reaction (EC CO_2_ RR) experiments were conducted in a conventional three-electrode system; a 1 mm thick Pt counter electrode, an Ag/AgCl (3.0 M KCl) reference electrode, and a V-Zn hybrid electrode (30 mm × 5 mm) working electrode. The electrodes were connected to a ZIVE SP1 compact type Potentiostat/Galvanostat (WonATech Co., Ltd., Seoul, Korea). Electrolytes were KHCO_3_, K_2_CO_3_, NaHCO_3_, Na_2_CO_3_, and KH_2_PO_4_/K_2_HPO_4_ buffer with the desired concentration. A volume of 50 mL electrolyte was taken in a 100 mL size glass cell. Pure CO_2_ gas (99.999%) was fully bubbled in the electrolyte and charged in a tightly closed EC cell. Amperometry experiments were conducted at a fixed applied potential (vs. Ag/AgCl) under dark, photoirradiation (365 nm, 3000 mW/cm^2^), and thermal irradiation (830 nm, 250 mW) conditions.

Photocatalytic CO_2_ RR experiment was conducted in a closed stainless-steel chamber with a quartz window (47 mm) on top for UVC (200–290 nm) light irradiation (5.94 mW/cm^2^) to a V-Zn disc inside (45 mm diameter). Before the experiment, 20 μL of deionized water was placed beside the sample and the chamber was fully flushed and charged with CO_2_ gas (99.999%). After that, UVC light was irradiated for 6 h on the disc sample through the quartz window.

After the EC and photocatalytic CO_2_ RR experiments, gas and liquid products were examined by gas chromatography (GC) and nuclear magnetic resonance spectroscopy (NMR), respectively. For gas products, 0.5 mL volume of gas was taken from the EC cell (or the photocatalyst closed reactor) and injected into a GC system (YL 6500 GC, Young In Chromass Co., Ltd., Seoul, Korea). Diverse gas products were separated using two different columns of 40/60 Carboxen-1000 (Sigma-Aldrich, St. Louis, MO, USA) and HP-Plot Q PT (Agilent Technologies, Inc., Santa Clara, CA, USA). The GC system was equipped with a thermal conductivity detector, a flame ionization detector, and a Ni catalyst methanizer assembly. For the analysis of liquid products, an NMR spectrometer (600 MHz FT-NMR, AVANCE III, Bruker Corp., Billerica, MA, USA) was employed with 0.5 mL electrolyte and 0.1 mL of DMSO/D_2_O (*v/v* = 1:20,000) internal standard.

## 3. Results and Discussion

**Figure 1:**
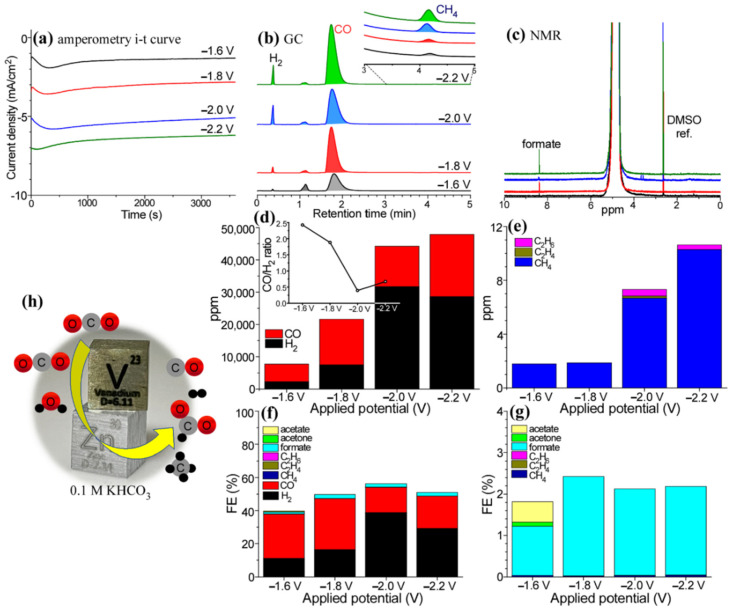
Amperometry i-t curves over V-Zn(H) hybrid electrodes at applied potentials of −1.6 V, −1.8 V, −2.0 V, and −2.2 V (vs. Ag/AgCl) (**a**). GC profiles after the amperometry tests (**b**). NMR spectra for liquid products after the amperometry tests (**c**). CO and H_2_ production amount with applied potentials (**d**). CH_4_, C_2_H_4_, and C_2_H_6_ amounts with applied potentials (**e**). Corresponding FEs including liquid products ((**f**) and (**g**), respectively). Scheme for CO_2_ reduction process over V-Zn hybrid material (**h**).

Figure 1a shows amperometry i-t curves over V/Zn(H) electrode obtained at various applied potentials of −1.6 V, −1.8 V, −2.0 V, and −2.2 V (vs. Ag/AgCl) for EC CO_2_ RR. The current density (CD) was increased with increasing the negative potential as expected. The CD was observed to be approximately 1.5 mA/cm^2^ and 6.5 mA/cm^2^ at −1.6 V and −2.0 V, respectively. The gas and liquid products were examined by taking GC and NMR profiles shown in Figure 1b,c, respectively. Major gas products were detected to be H_2_ and CO. A minor product includes CH_4_. A major liquid product was observed to be mainly formate. Other NMR signals were in impurity levels that were not discussed in detail. In Figure 1d, CO production was higher than H_2_ production at a lower negative applied potential; 2266 ppm and 28,643 ppm at −1.6 V and −2.0 V, respectively. The CO/H_2_ (syngas) ratio was estimated to be 2.5 at −1.6 V and decreased with increasing the negative potential; this reflects that the syngas ratio could be controlled from 2.5 to 0.4 by applying applied potential. In Figure 1e, CH_4_ was very meaningfully produced and increased with applied potential; 1.8 ppm at −1.6 V and 10.3 ppm at −2.0 V. Although their amounts were only below 1 ppm the productions of C_2_H_4_ and C_2_H_6_ were not negligible. Figure 1f shows the Faradaic efficiency (FE) of the detectable products with applied potentials. The FE of H_2_ was maximized to be 38.8% at −2.0 V, but the FE of CO was minimized to 15.4% at this applied potential. The maximum FE of CO was 30.9% at −1.8 V. The FE of formate was observed to be between 1.2% (at −1.6 V) and 2.4% (at −1.8 V). The FEs of other products were small; e.g., lower than 0.04% for CH_4_. For minor liquid products at −1.6 V, acetate and acetone were weakly detectable with FEs of 0.5% and 0.1%, respectively; however, these were not clearly observed at higher applied potentials. In Figure 1h, the image depicts the CO_2_ reduction process with major products of CO, formate, and CH_4_ over V-Zn hybrid material.

**Figure 2:**
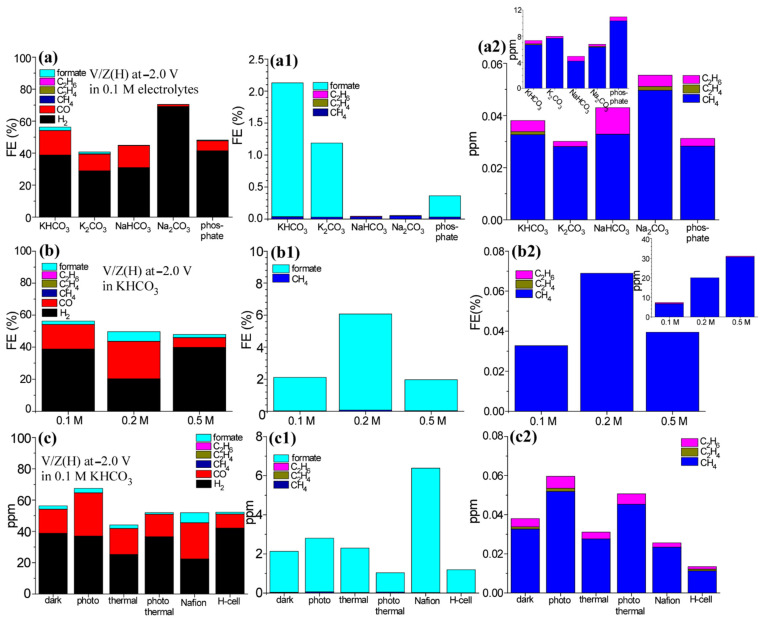
FEs of CO_2_ RR products over V-Zn(H) electrode tested in various electrolytes (**a**,**a1**,**a2**). FEs tested in KHCO_3_ electrolyte with different concentrations of 0.1 M, 0.2 M, and 0.5 M (**b**,**b1**,**b2**). FEs in 0.1 M KHCO_3_ electrolyte tested under dark, photoirradiation, thermal irradiation, photothermal irradiation, Nafion-treated electrode, and H-cell condition (**c**,**c1**,**c2**).

The V/Zn(H) electrode was further tested in diverse different electrolytes of KHCO_3_, K_2_CO_3_, NaHCO_3_, Na_2_CO_3_, and KH_2_PO_4_/K_2_HPO_4_ buffer with a concentration of 0.1 M (Figure 2(a,a1,a2)). All the FEs were observed to be highly dependent on the electrolyte. The FEs of H_2_ and CO were all decreased in K_2_CO_3_ (28.9% and 10.4%, respectively) and NaHCO_3_ (30.9% and 13.8%, respectively) electrolytes, compared with those (38.8% and 15.4%, respectively) in 0.1 M KHCO_3_ electrolyte (Figure 2a). The FE of H_2_ was increased in Na_2_CO_3_ (69.2%) and KH_2_PO_4_/K_2_HPO_4_ buffer (41.5%) electrolytes, but the FE of CO was decreased in the electrolytes with FEs of 1.2% and 6.3%, respectively. The FEs of formate were all decreased to 1.2%, 0%, 0%, and 0.3% in K_2_CO_3_, NaHCO_3_, Na_2_CO_3_, and KH_2_PO_4_/K_2_HPO_4_ buffer, respectively (Figure 2(a1)). Formate was not produced in NaHCO_3_ and Na_2_CO_3_ electrolytes; this indicates that Na^+^ plays a negative role in formate production, due to interaction between solvated cations and the adsorbed species [30]. CH_4_ production was commonly dependent on H_2_ production (Figure 2(a2)). The FE of CH_4_ was maximized to be 0.05% in 0.1 M Na_2_CO_3_ electrolyte where the FE of H_2_ was shown to be maximum. The production of C_2_H_6_ was higher than that of C_2_H_4_ and was observed to be between 0.002% (corresponding to 0.3 ppm) and 0.01% (corresponding to 0.7 ppm).

The V/Zn(H) electrode was tested in different concentrations of 0.1 M, 0.2 M, and 0.5 M KHCO_3_ electrolytes (Figure 2(b,b1,b2)). In 0.2 M concentration, CO_2_ RR performance was significantly improved, compared with the result in 0.1 M concentration. The FEs of CO and formate were dramatically increased to 23.2% by 1.5× and 6.0% by 2.9×, respectively, compared with those in 0.1 M concentration. The FE of H_2_ was instead decreased from 15.4% to 6.1%. In 0.5 M concentration, the FEs of CO and formate were observed to be only 6.1% and 1.9%, respectively, much smaller than those in 0.1 M and 0.2 M concentrations (Figure 2(b,b1)). The CH_4_ production amount (ppm) was linearly increased with increasing the concentration; 6.7 ppm, 20.0 ppm, and 30.7 ppm in 0.1 M, 0.2 M, and 0.5 M, respectively (inset in Figure 2(b2)). The FE of CH_4_ was maximized to be 0.07% in 0.2 M (Figure 2(b2)). C_2_H_4_ and C_2_H_6_ were not detected at all in these conditions.

The V/Zn(H) electrode was further tested at −2.0 V in 0.1 M KHCO_3_ electrolyte under other various conditions of dark, photoirradiation (365 nm), thermal irradiation (830 nm), photothermal irradiation (365 nm + 830 nm), Nafion-treated electrode, and H-cell condition (Figure 2(c,c1,c2)). The FE of CO was distinctly increased to 27.6% by 1.8× under photoirradiation (365 nm), compared with that under dark conditions (Figure 2c). The increase in CO under photoirradiation was in good consistent with the literature [3]. The FE was also increased to 23.2% by 1.5× when the electrode was treated with Nafion. The FE in the other conditions showed no dramatic effect and decreased to 9.0% in the H-cell condition. In the H-cell condition, the FE of H_2_ was instead increased to 42.1%. For the formate production (Figure 2(c1)), the FE was somewhat increased under photoirradiation and thermal irradiation conditions. The FE of formate was more significantly increased to 6.4% by 3.0×, compared with that under dark conditions. For the FE of CH_4_ production (Figure 2(c2)), CH_4_ production was enhanced under photoirradiation but decreased in Nafion-treated electrode and H-cell conditions. On the basis of the results, it was concluded that the CO_2_ RR performance was highly dependent on the experimental conditions.

**Figure 3:**
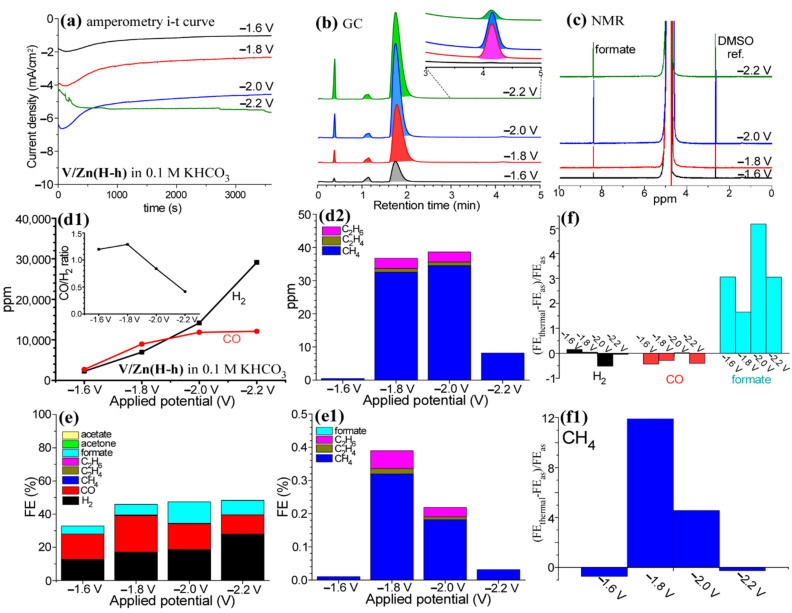
Amperometry i-t curves over V-Zn(H-h) electrode at various applied potentials of −1.6 V, −1.8 V, −2.0 V, and −2.2 V (vs. Ag/AgCl) in 0.1 M KHCO_3_ electrolyte (**a**). Corresponding GC (**b**) and NMR (**c**) profiles for gas and liquid products, respectively. Measured H_2_ and CO product amounts (**d1**). CH_4_, C_2_H_4_, and C_2_H_6_ amounts (**d2**). Corresponding FEs of gas and liquid products (**e**,**e1**). (FE_V/Zn(H-h)_−FE_V/Zn(H)_)/FE_V/Zn(H)_ ratios for H_2_, CO, and formate productions (**f**). FE_V/Zn(H-h)_−FE_V/Zn(H)_)/FE_V/Zn(H)_ ratios for CH_4_ production (**f1**).

The V/Zn(H) electrode interface was modified by thermal annealing at 400 °C. The consequent V/Zn(H-h) electrode was tested in 0.1 M KHCO_3_ electrolyte with various applied potentials of −1.6 V, −1.8 V, −2.0 V, and −2.2 V (vs. Ag/AgCl). In the amperometry i-t curves (Figure 3a), the final CD value at 3600 s was increased with increasing the potential; 1.0 mA/cm^2^, 2.3 mA/cm^2^, 4.6 mA/cm^2^, and 5.7 mA/cm^2^ at −1.6 V, −1.8 V, −2.0 V, and −2.2 V (vs. Ag/AgCl), respectively; however, for the initial and final CD values the i-t curve showed different behaviour at an applied potential of −2.2 V; this indicates that the V/Zn(H-h) electrode was oxidized after thermal annealing and more significantly altered during the initial EC at the highest applied potential. The gas and liquid products were examined by GC and NMR profiles shown in Figure 3b,c, respectively. Major gas products were detected to be H_2_ and CO that were increased with applied potential. Minor products include CH_4_, C_2_H_4_, and C_2_H_6_ (Figure 3b). A major liquid product was formate and others were unassignable (Figure 3c).

The H_2_ production was drastically increased with increasing potential; 2250 ppm and 29,115 ppm at −1.6 V and −2.2 V, respectively (Figure 3(d1)). CO production was sharply increased from 2693 ppm to 8952 ppm when the potential was increased from −1.6 V and −1.8 V; however, the CO production showed a sluggish increase above −1.8 V. As a consequence, CO production amount was higher than H_2_ production at −1.6 V and −1.8 V, but lower at −2.0 V and −2.2 V. In other words, the CO/H_2_ ratio was estimated to be 1.2 and 1.3 at −1.6 V and −1.8 V, respectively, but 0.84 and 0.41 at −2.0 V and −2.2 V, respectively. The syngas ratio was consequently tuned from 1.3 to 0.4. CH_4_ was only detected to be 0.4 ppm at −1.6 V, but detected to be 32–35 ppm at −1.8 V and −2.0 V. At a higher potential of −2.2 V, the CH_4_ amount was decreased to 8.1 ppm. As shown in Figure 3e, the FE of H_2_ was increased with applied potential and reached 28.0% at −2.2 V. The FE of CO at −1.8 V was maximized and observed to be 22.0%. The FE of CO decreased to 15.6% and 11.6% at higher potentials of −2.0 V and −2.2 V, respectively. The FE of formate was shown to be a maximum of 12.9% at −2.0 V. in Figure 3(e1), the FEs of CH_4_, C_2_H_4_, and C_2_H_6_ were observed to be maxima at −1.8 V.

The (FE_V/Zn(H-h)_−FE_V/Zn(H)_)/FE_V/Zn(H)_ ratios were estimated, where FE_V/Zn(H-h)_ is the FE of the thermal-treated sample and FE_V/Zn(H)_ is the FE of the as-prepared sample at an applied potential. In Figure 3f, the ratios for H_2_ and CO commonly showed negative values, indicating that the thermal-treated sample showed smaller FEs for H_2_ and CO productions. For the formate production, the ratios showed high values between 1.6 and 5.2, indicating that the thermal-treated sample showed higher performance for formate production. In Figure 3(f1), the ratios for CH_4_ production showed high positive values at −1.8 V and −2.0 V, but small negative values at −1.6 V and −2.2 V.

**Figure 4:**
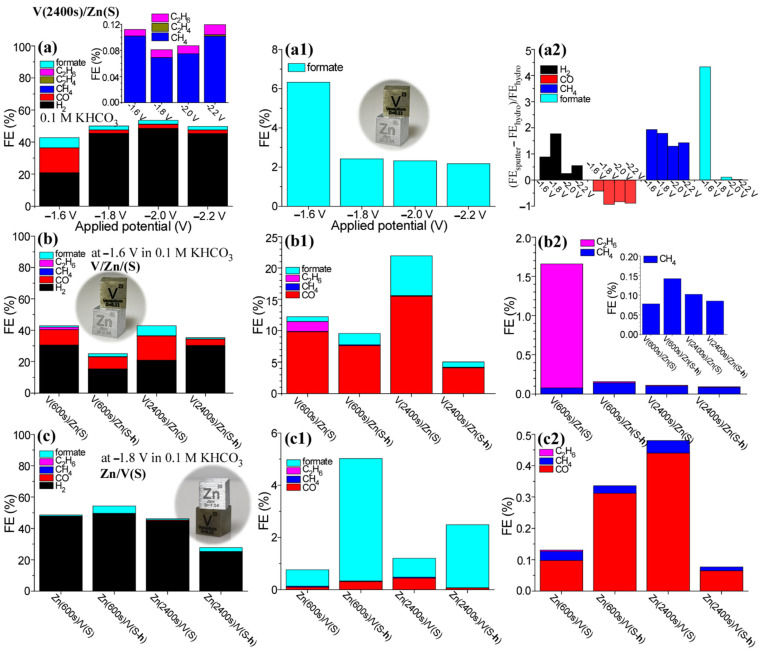
FEs of gas and liquid products over V/Zn(S) electrode at various applied potentials of −1.6 V, −1.8 V, −2.0 V, and −2.2 V (vs. Ag/AgCl) in 0.1 M KHCO_3_ electrolyte (**a**,**a1**). (FE_V/Zn(S)_-FE_V/Zn(H)_)/FE_V/Zn(H)_ ratios for H_2_, CO, CH_4_, and formate productions (**a2**). FEs of gas and liquid products over V(600 s)/Zn(S), V(600 s)/Zn(S-h), V(2400 s)/Zn(S), V(2400 s)/Zn(S-h) at −1.6 V in 0.1 M KHCO_3_ electrolyte, and over V(2400 s)/Zn(S) at −1.8 V (**b**,**b1**,**b2**). FEs of gas and liquid products over Zn(600 s)/V(S), Zn(600 s)/V(S-h), Zn(2400 s)/V(S), Zn(2400 s)/V(S-h) at −1.8 V in 0.1 M KHCO_3_ electrolyte (**c**,**c1**,**c2**).

Another V/Zn interface was prepared by sputter-deposition of V on Zn foil support and also inversely sputter deposition of Zn on V foil support. Figure 4(a,a1) shows FEs of detectable gas and liquid products for as-sputtered V(2400 s)/Zn(S) electrode with various applied potentials of −1.6 V, −1.8 V, −2.0 V, and −2.2 V (vs. Ag/AgCl). The FEs of H_2_, CO, and formate were measured to be 20.9%, 15.5%, and 6.3% at −1.6 V, respectively (Figure 4a). The FE of H_2_ was increased at higher potentials, but those of CO and formate were decreased at higher potentials. The FE of formate at −1.6 V was approximately three times higher than those at higher applied potentials (Figure 4b). The FEs of CH_4_ and C_2_H_6_ are shown in the inset of Figure 4a. The (FE_V/Zn(S)_−FE_V/Zn(H)_)/FE_V/Zn(H)_ ratios were estimated (Figure 4(a2)), where FE_V/Zn(S)_ is the FE of the sample by sputter deposition and FE_V/Zn(H)_ is the FE of the sample by hydrothermal method at an applied potential. The ratios for H_2_, CH_4_, and formate showed positive value, indicating that the V/Zn electrode by sputter deposition method showed higher performances in H_2_, CH_4_, and formate productions at all applied potentials, compared with those over the V/Zn electrode by hydrothermal method. On the other hand, the ratio for CO production showed negative values at all applied potentials, indicating that the V/Zn electrode by sputter deposition showed poor performance than the V/Zn electrode by hydrothermal method.

The thickness of V and thermal treatment (400 °C for 1 h) effects were examined for the V/Zn(S) electrodes prepared by sputter deposition. As shown in Figure 4b, the FE of H_2_ was increased upon thermal treatment for V(600 s)/Zn electrode. On the other hand, the FE of H_2_ was increased upon thermal treatment for V(2400 s)/Zn electrode. For the FE of CO (Figure 4(b1)), the thermal treatment showed a negative effect for both V(600 s)/Zn(S) and V(2400 s)/Zn(S) electrodes. The as-sputtered V(600 s)/Zn(S) electrode showed an FE of 9.8% for CO, but decreased to 7.6% upon thermal treatment. The as-sputtered V(2400 s)/Zn(S) electrode showed an FE of 15.5% for CO, but decreased to 4.1% upon thermal treatment. For the FE of formate, the V(600 s)/Zn(S) electrode showed enhancement upon thermal treatment from 0.8% to 1.8%, but the V(2400 s)/Zn(S) electrode showed diminishment upon thermal treatment from 6.3% to 0.8%. Interestingly, the FE of C_2_H_6_ showed a large enhancement with a FE of 1.6%, compared with other samples (Figure 4(b2)). In addition, the FE was larger than that of CH_4_. Although it is not clear whether the experimental observation was meaningful. The FE of CH_4_ showed the highest FE of 0.14% for the V(600 s)/Zn(S-h) electrode (inset of Figure 4(b2)).

When Zn was used as a support material the EC CO_2_ RR showed higher FEs for CO and formate, as discussed above. Inversely, the Zn/V interface was prepared by sputter-deposition of Zn on V foil support and tested for EC CO_2_ RR. As shown in Figure 4c, H_2_ production was major, but CO production was dramatically diminished. The FE of formate was distinctly enhanced upon thermal treatment at 400 °C for 1 h. The FE of formate for Zn(600 s)/V(S) was increased from 0.6% to 4.7% (Figure 4(c1)). The FE of formate for Zn(2400 s)/V(S) was increased from 0.7% to 2.4%. The FE of CO was much lower than 1% (Figure 4(c2)). The FE of CO for Zn(600 s)/V(S) was increased from 0.1% to 0.3%. The FE of CO for Zn(2400 s)/V(S) was decreased from 0.4% to 0.06%. The FE of CO and CH_4_ showed the highest for the as-sputtered Zn(2400 s)/V(S) electrode; this indicates that Zn played a more important role in EC CO_2_ RR.

Generally, the total FE(%) was observed to be less than 100%. Several reasons appeared to be involved; the electrochemical current producing undetectable products, non-Faradaic current occurring at the metal oxide electrode surface, dissolution of gas products in electrolyte, and surface reduction reaction current of V-Zn oxides [3].

**Figure 5:**
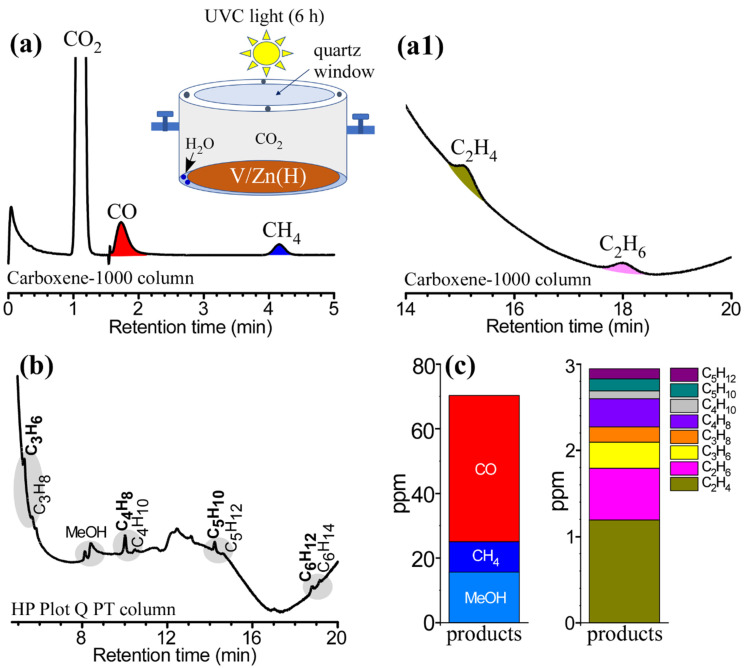
GC profiles separated by Carboxene-1000 column (**a**,**a1**) after photocatalytic CO_2_ RR for 6 h under UVC irradiation. Inset is a schematic of a closed stainless-steel reactor. GC profile separated by HP Plot Q PT column (**b**). Consequent photocatalytic CO_2_ RR product amounts (**c**).

For direct comparison with the EC process, photocatalytic CO_2_ RR was tested for a selected sample of V/Zn(H) disc (Figure 5). The experiment was conducted in the gas (CO_2_ and H_2_O)-solid (catalyst) mode as depicted in the inset of Figure 5a [2,4]. Photocatalytic CO_2_ RR experiments over V-Zn hybrids in the literature have commonly been conducted in the liquid (bulk H_2_O with dissolved CO_2_)-solid (dispersed powder) mode [14,15,16,17]. Gas products were mainly detected because of the gas-phase mode. CO, CH_4_, C_2_H_4_, and C_2_H_6_ were clearly detected in the GC profiles separated by the Carboxene-1000 column (Figure 5(a,a1)). More interestingly, although the amounts were small Hydrocarbons (C_n_H_2n_ and C_n_H_2n+2_, where n = 3–6) were detected in the GC profile separated by HP Plot Q PT column (Figure 5b), including CH_3_OH (MeOH). The production amounts of CO, CH_4_, and MeOH were measured to be 45.2 ppm, 9.4 ppm, and 15.6 ppm (Figure 5c). CO was more produced than CH_4_, in good with the literature using a similar catalyst system of V_2_O_5_/ZnV_2_O_6_ nanosheets [14]. The total amounts of C_2-5_ reached to approximately 3 ppm. In the photocatalytic CO_2_ RR, alkene (C_n_H_2n_) was produced more than alkanes (C_n_H_2n+2_). The C_2_H_4_/C_2_H_6_ ratio was observed to be 2.0. In the EC CO_2_ RR, the C_2_H_6_ production was more dominant than C_2_H_4_ (e.g., 6.7 ppm vs. 0.2 ppm at −2.0 V over V/Zn(H) electrode). The alkene/alkane ratios were estimated to be 1.7, 3.6 and 1.2 for C_3_, C_4_, and C_5_ hydrocarbons, respectively. The long-chain formation was understood by C-C coupling in the conventional Fisher-Tropsch synthesis [25,26], further discussed below.

**Figure 6:**
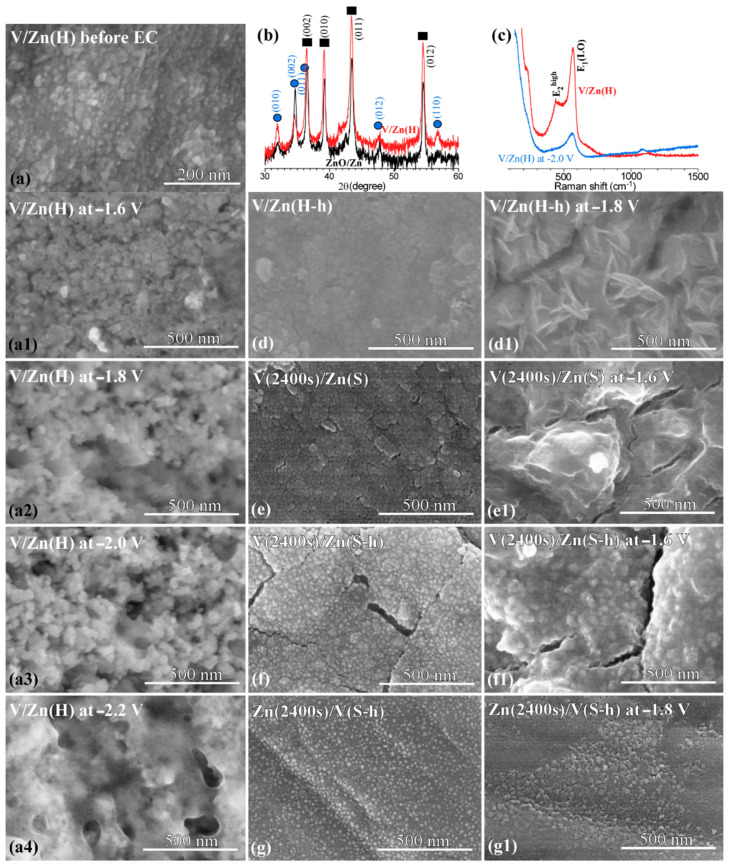
SEM images of V/Zn(H) electrode before (**a**) and after EC at various applied potentials (**a1**–**a4**). XRD profiles (**b**) of V/Zn(H) and ZnO/Zn (reference). Raman spectra of V/Zn(H) electrode before and after EC at −2.0 V (**c**). V/Zn(H-h) (**d**,**d1**), V(2400 s)/Zn(S) (**e**,**e1**), V(2400 s)/Zn(S-h) (**f**,**f1**), and Zn(2400 s)/V(S-h) (**g**,**g1**) electrodes before and after EC.

Figure 6 displays SEM images (a, a1, a2, a3, and a4) of V/Zn(H) electrodes before and after EC at various applied potentials of −1.6 V, −1.8 V, −2.0 V, and −2.2 V (vs. Ag/AgCl); it was commonly observed that the surface morphology became changed and the surface became more drastically changed with increasing the applied potential. The surface reconstruction has commonly been reported during EC CO_2_ reduction at negative applied potentials, especially for oxide materials due to the reduction of surface oxide species [21,31]. XRD profiles (Figure 6b) were obtained for V/Zn(H) electrode and a ZnO/Zn reference electrode. XRD profiles of the two different samples were quite similar. Strong XRD patterns (closed squares, ■) were commonly observed at 2θ = 36.7°, 39.4°, 43.7°, and 54.7°, attributed to the (002), (010), (011), and (012) crystal planes of metallic Zn [32,33,34]; these signals were from the Zn support material. The other XRD signals (closed circles, ●) were also commonly observed at 2θ = 32.0°, 34.7°, 36.5°, 47.7°, and 56.9°, attributed to the (010), (002), (011), (012), and (110) crystal planes of hexagonal phase wurtzite ZnO [21,27,32]; this indicates that ZnO phase was commonly formed during the hydrothermal synthesis using metallic Zn support. No significant XRD profiles of V oxide and Zn-V oxides were observed, indicating that these species were ultrathin and/or amorphous; however, V species were clearly observed by XPS, discussed below. In Figure 6c, the Raman profile of the V/Zn(H) electrode showed mainly characteristics of ZnO [31]. The peaks around 435 cm^−1^ and 570 cm^−1^ were assigned to E_2_ high and E_1_(LO) modes, respectively [31]. The peaks were weakened after EC, due to surface reduction of V-Zn oxide species. SEM images were obtained for other demonstrated samples of V/Zn(H-h), V(2400 s)/Zn(S), V(2400 s)/Zn(S-h), and Zn(2400 s)/V(S-h) before and after EC in Figure 6(d,d1),(e,e1),(f,f1),(g,g1), respectively. The morphologies were different from those of V/Zn(H) electrodes as expected for different electrode materials.

**Figure 7:**
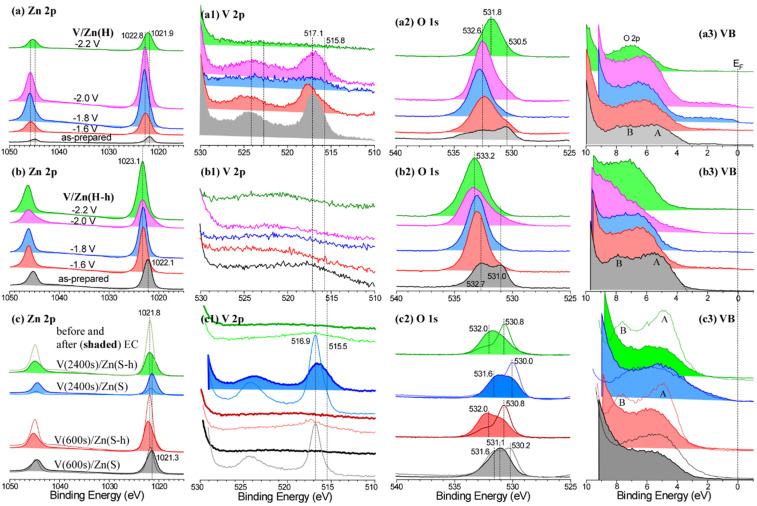
Zn 2p, V 2p, O 1s, and VB XPS profiles for V/Zn(H) electrode before and after tested at various applied potentials ((**a**–**a3**), respectively), V/Zn(H-h) electrode before and after tested at various applied potentials ((**b**–**b3**), respectively), and V(600 s)/Zn(S), V(600 s)/Zn(S-h), V(2400 s)/Zn(S), V(2400 s)/Zn(S-h) electrodes before and after EC (shaded area) at −1.6 V in 0.1 M KHCO_3_ electrolyte ((**c**–**c3**), respectively).

XPS spectra were obtained to examine oxidation states, stability before and after EC, and differences in surface electronic structures before and after thermal treatment for the interface engineered V/Zn electrodes. Figure 7(a–a3) display Zn 2p, V 2p, O 1s, and VB XPS profiles, respectively for V/Zn(H) electrodes before and after EC CO_2_ RR at −1.6 V, −1.8 V, −2.0 V, and −2.2 V. For the Zn 2p XPS of as-prepared V/Zn(H) electrode before EC (Figure 7a), Zn 2p_3/2_ and Zn 2p_1/2_ peaks were observed at binding energies (BEs) of 1021.9 eV and 1044.9 eV, respectively with a spin-orbit (S-O) splitting energy of 23.0 eV; this is plausibly due to Zn(II) oxide species [14,35,36,37,38]. In Figure 7(a1), V 2p_3/2_ peaks could be resolved into two peaks at 517.1 eV (major) and 515.8 eV (shoulder), attributed to V^5+^ and V^4+^, respectively [6,7,14,37]. The V 2p_3/2_ peak at 517.1 eV was predominant. For the O 1s XPS of the as-prepared V/Zn(H) electrode sample (Figure 7(a2)), two broad peaks were observed at 530.5 eV and 532.6 eV, commonly attributed to lattice oxygen (O_L_) of Zn-V oxide species and surface oxygen species (e.g., O_ad_: OH/H_2_O, OH^−^, and defects), respectively [7,14,15,39,40]. For the corresponding valence band (VB) profile (Figure 7(a3)), the VB edge was positioned at 3.0 eV below the Fermi level. The corresponding band gap was observed to be 3.1 eV (measured by UV-visible absorption spectrometer), indicating that the conduction band (CB) edge is positioned near the Fermi level. Two broad features were seen around 5.0 eV (A) and 8.0 eV, attributed to hybridizations of O 2p/Zn 3d/partially Zn 4p states and O 2p/Zn 3d/Zn 4s states, respectively [41]; it appears that O 2p state is highly contributed in the VB structure near the Femi level [13].

For the Zn 2p XPS after EC CO_2_ RR at −1.6 V, −1.8 V, and −2.0 V, the Zn 2p_3/2_ peak position was observed at a higher BE position of 1022.8 eV. V 2p peaks were somewhat weakened, but their BE positions were slightly altered (Figure 7(a1)). More clearly, the O 1s peak for O_L_ was weakened while the O 1s for O_ad_ became dominant. In the VB region, A and B peaks appeared to be merged one peak centred around 6 eV; it appears that the surface state changed to more like Zn(II)/V-OH species [35]. Upon applying the highest potential of −2.2 V, the Zn 2p_3/2_ peak was again shifted to a lower BE position of 1022.1 eV. The corresponding V 2p peak disappeared, indicating that the surface V species were dissolved and diffused into the bulk electrolyte during EC. The O 1s and VB XPS peaks became critically different from others, due to mainly surface states of V-free reduced ZnO/Zn(OH)_2_ [35,36,37,38].

For the XPS of thermal treated electrode of V/Zn(H-h) (Figure 7(b–b3)), Zn 2p_3/2_ and Zn 2p_1/2_ peaks were observed at binding energies (BEs) of 1022.1 eV and 1045.2 eV, respectively with a S-O splitting energy of 23.1 eV (Figure 7b); this is plausibly due to Zn(II)/V oxide species [35,36,37]. For the Zn 2p XPS after EC CO_2_ RR at −1.6 V, −1.8 V, −2.0 V, and −2.2 V, the Zn 2p_3/2_ peak position was observed at a higher BE position of 1023.1 eV, plausibly due to Zn(II)/V-OH species [35]. V 2p peaks were not seen (Figure 7(b1)), due to diffusion of V into the bulk state upon thermal treatment. For the O 1s XPS of V/Zn(H-h), two broad peaks were seen at 531.0 eV and 532.7 eV, attributed to lattice oxygen (O_L_) of Zn-V oxide species and surface oxygen species (e.g., O_ad_: OH/H_2_O, OH^−^, and defects), respectively, as mentioned above [7,39,40]. After EC CO_2_ RR at −1.6 V, −1.8 V, −2.0 V, and −2.2 V, a broad peak around 533.2 eV became dominant, attributed to an increase in surface oxygen species such as OH. In the VB XPS profiles (Figure 7(b3)), two broad features (A and B) became more distinct and appeared to be merged one after EC CO_2_ RR, as mentioned above.

For the XPS profiles of sputtered V on Zn foil support before and after thermal treatment (Figure 7(c–c3)), a Zn 2p_3/2_ peak was observed at 1021.8 eV, due to the more metallic nature of Zn. The BE position showed no critical change after thermal treatment, due to the protection of overlayer V. After EC CO_2_ RR at −1.6 V, the BE position of the Zn 2p_3/2_ peak was commonly shifted to 1021.3 eV. The Zn 2p XPS profiles of V(600 s)/Zn(S) and V(2400 s)/Zn(S) were qualitatively similar before and after EC. A V 2p_3/2_ peak was dominantly seen at 516.9 eV, attributed to V^5+^ of V_2_O_5_ [6,7,37]. The V 2p of V(2400 s)/Zn(S) was stronger than that of V(600 s)/Zn(S) as expected. V 2p peaks became disappeared (Figure 7(c1)), due to diffusion of V into bulk state upon thermal treatment. For the V 2p XPS after EC, the peak intensity was quite weakened, especially for V(600 s)/Zn(S); however, the V 2p signal of V(2400 s)/Zn(S) was quite stable and broadened after EC. The BE distribution around 515.5 eV was due to V^4+^. The O 1s XPS profiles of V(600 s)/Zn(S) and V(2400 s)/Zn(S) were also qualitatively similar before and after EC (Figure 7(c2)). The O 1s XPS peaks of V(600 s)/Zn(S) and V(2400 s)/Zn(S) before thermal treatment were observed around 530.2 eV (530.0 eV) and 531.6 eV, due to lattice oxygen (O_L_) and surface oxygen species, respectively. After thermal treatment for V(600 s)/Zn(S-h) and V(2400 s)/Zn(S-h), two O 1s peaks were observed around 530.8 eV and 532.0 eV, attributed to lattice oxygen (O_L_) and surface oxygen species, respectively. After EC CO_2_ RR at −1.6 V, the lattice O 1s peak was weakened while the surface O 1s peak became stronger, as discussed above. For the VB profiles, a broad feature was commonly seen around 6 eV, attributed to hybridized O 2p states with Zn/V. The thermally treated sample of V(600 s)/Zn(S-h) and V(2400 s)/Zn(S-h) showed more clearly two features around 5.0 (A) and 8.0 eV (B), as mentioned above; these two merged one after EC, also discussed above.

**Figure 8:**
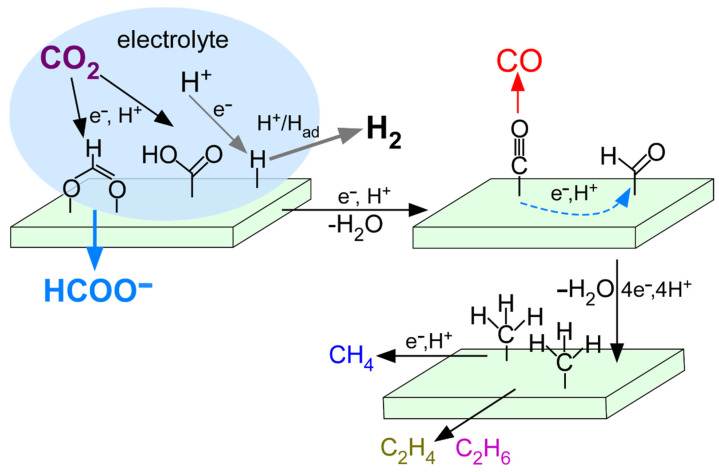
Proposed CO_2_ RR mechanism on the basis of the products.

On the basis of the products tested over diverse electrode samples, the CO_2_ RR mechanism was discussed below and depicted in Figure 8. In an electrolyte, H^+^, electrolyte cations/anions, and CO_2_ are commonly present. In CO_2_ RR, H^+^ is consumed for producing organic compounds, and negatively and competitively used for H_2_ production via H^+^ + e^−^ → H_ad_ followed by H_ad_ + H_ad_ → H_2_ or H_ad_ + H^+^ + e^−^ → H_2_ [3,4,5,39,40]. CO_2_ is adsorbed on an electrode surface commonly with two different bindings via CO_2_ + H^+^ + e^−^ → HCOO_ad_ or HOOC_ad_ [3,4]. The O or C of CO_2_ is hybridized with surface metal orbitals as depicted in Figure 8. When HCOO_ad_ is formed it may release into bulk electrolyte as formate. The hybridization forming HCOO_ad_ was observed to be enhanced by thermal annealing of both V/Zn(H) and Zn/V(S). In other words, formate production was further improved by a more V-Zn hybridized state. The fate HOOC_ad_ is transformed into OC_ad_ via HOOC_ad_ + H^+^ + e^−^ → OC_ad_ + H_2_O [3,4]. The OC_ad_ is then released into free CO or converted into other surface species. For the productions of minor CH_4_, C_2_H_4_, and C_2_H_6_, it requires a surface reaction process of OC_ad_ + H^+^ + e^−^ → _ad_CHO. The _ad_CHO species proceed surface _ad_CH_2_ and _ad_CH_3_ via _ad_CHO + 3H^+^ + 3e^−^ → _ad_CH_2_ + H_2_O and _ad_CHO + 4H^+^ + 4e^−^ → _ad_CH_3_ + H_2_O, respectively [3,4]. The CH_3_ associates with H to form CH_4_ via CH_3_ + H → CH_4_ or CH_3_ + H^+^ + e^−^ → CH_4_ [42,43]. The surface CH_x_ (x = 2, 3) may couple to generate free C_2_H_4_ and C_2_H_6_. The productions of CH_4_, C_2_H_4_, and C_2_H_6_ were significantly increased when V-Zn hybridization was increased by thermal annealing treatment. When the desorption of CO is easy the productions of CH_4_, C_2_H_4_, and C_2_H_6_ are expected to be reduced, or vice versa.

In electrochemical CO_2_ reduction, it is not easy to determine different active sites involved because of diverse surface species, electrode surface reconstruction, and the topmost element diffusion into a bulk electrolyte during electrochemical reaction [44,45]. For the different active sites, it needs to determine hydrogen adsorption, CO adsorption by the stripping method, and formate adsorption by the oxidation peak analysis [44,45]. For these V-Zn hybrids, the surface electronic structures are altered by different V/Zn ratios, the amounts and active site species become different, and thereby CO_2_ reduction performances are expected to be different. In the present study, two different d-block transition metal elements of V and Zn were used to examine EC CO_2_ reduction performances. To increase selectivity for a desired product, it is also necessary to employ different synthesis methods and other metal elements for hybrid systems; for example, Cu for C-C compounds, Au for CO, and p-block elements for formate [46,47,48,49].

For the CO_2_ RR products by photocatalysis, CH_3_OH and meaningful amounts of C_3-5_ compounds were newly detected. CO, CH_4_, C_2_H_4_, and C_2_H_6_ were common products as observed in electrocatalysis. In addition, the alkenes were more produced than the alkanes. For example, as discussed above, C_2_H_4_ was more produced than C_2_H_6_, unlike in electrocatalysis and conventional F-T synthesis. The alkene/alkane ratio has commonly been determined by the nature of a catalyst and the surface H/CO ratio at an experimental condition [50]. To confirm the alkene preference, we have also tested photocatalytic CO_2_ RR experiments over other transition metal elements and found similar results (not shown here). On the basis of these results, intuitively, because the neighbouring surface H in photocatalysis is much less than in electrocatalysis (and conventional high-pressurized conventional F-T synthesis) unsaturated alkenes are expected to be more produced in photocatalysis. Otherwise, the V-Zn surface may not be active for hydrogenation reaction; it needs further study to confirm the mechanism.

CH_3_OH is expected to be produced via _ad_CHO + 3H_ad_ → CH_3_OH (g). For the production of long-chain C_3-5_ compounds, C-C coupling can be understood by (1) coupling of surface CH_x_ and (2) CO insertion mechanism proposed in the conventional Fischer–Tropsch (F-T) synthesis [25,26]. In the coupling of surface CH_x_, the coupling of CH_x_ occurs via CH_3_(CH_2_)_x_C_ad_H_2_ + CH_x_ → CH_3_(CH_2_)_x+1_C_ad_H_2_ followed by association with surface H to liberate alkenes and alkanes. The CO insertion mechanism occurs via CH_3_(CH_2_)_x_C_ad_H_2_ + CO + H_ad_ → CH_3_(CH_2_)_x+1_C_ad_H_2_ + HO_ad_. As a consequence, longer chain hydrocarbons were formed.

## 4. Conclusions

In summary, V-Zn hybrids were prepared by hydrothermal (H) and sputter-deposition (S) methods using a Zn foil support. For EC CO_2_ RR over V/Zn(H) electrode in 0.1 M KHCO_3_, CO (FE = 15–31%), H_2_ (FE = 11–39%), and formate (FE = 1.1–2.4%) were mainly produced with minor CH_4_, C_2_H_4_, and C_2_H_6_. Syngas (CO/H_2_) ratio was tuned from 2.5 to 0.4 by applying applied potential from −1.6 V to −2.2 V (vs. Ag/AgCl). The maximum FE of CO was 30.9% at −1.8 V in 0.1 M KHCO_3_ electrolyte. The KHCO_3_ electrolyte showed the best performance in CO and formate production, compared with other demonstrated electrolytes. In 0.2 M KHCO_3_ electrolyte, the FEs of CO, CH_4_, and formate were increased by 1.5×, 2.3×, and 2.9×, respectively, compared with those in 0.1 M KHCO_3_ electrolyte. CO and formate were further increased under photoirradiation conditions and by using a Nafion-treated electrode. For EC CO_2_ RR over V/Zn(H-h) electrode, formate production was dramatically increased by 160–520%, compared with those over the V/Zn(H) electrode, understood by an increased hybridization of V and Zn. CH_4_ production was increased by 450–1200%.

For EC CO_2_ RR over V/Zn(S) electrode in 0.1 M KHCO_3_, the FEs of CO and formate at −1.6 V were much higher than those at higher applied potentials. CH_4_ and formate were produced more in the V/Zn(S) electrode, compared with those in V/Zn(H) electrode. CO production was negated in the V/Zn(S) electrode by sputter deposition. CO production was negated by thermal treatment of the V/Zn(S) electrode. For Zn/V(S) electrodes, when V support was used CO production was drastically diminished and the FE of CO was much lower than 1%. The FE of formate was also lower than 1%. When Zn/V(S) electrode was thermally treated the FE of formate was increased to 2–5%, due to an increased hybridization of V and Zn; this is in good consistency with the V/Zn(H) electrode. The EC CO_2_ RR mechanism was proposed by two different initial bindings via CO_2_ + H^+^ + e^−^ → HCOO_ad_ or HOOC_ad_. The hybridization between surface V/Zn and C and O may determine the production of CO, formate, and C_2_ compounds.

In photocatalysis over V/Zn(H), CH_3_OH, CO, and CH_4_ were mainly produced. Meaningful amounts of C_2-6_ hydrocarbons were first observed to be produced. The long chain formation was understood by the coupling of surface CH_x_ and CO insertion mechanism in the F-T synthesis process. Alkenes were more produced than alkanes unlike in electrocatalysis and conventional F-T synthesis.

Overall, the present unique results on electrocatalysis and photocatalysis over interface engineered V-Zn hybrid materials provide deeper information on the development of V-Zn hybrid materials producing value-added carbon products from CO_2_ and H_2_O by electrocatalyst as well as photocatalysis for energy and the environment.

## Data Availability

The data presented in this study are available in the article.
